# Dispersal and Transmission of Avian Paramyxovirus Serotype 4 among Wild Birds and Domestic Poultry

**DOI:** 10.3389/fcimb.2017.00212

**Published:** 2017-05-26

**Authors:** Renfu Yin, Pingze Zhang, Xinxin Liu, Yanyu Chen, Zhi Tao, Lili Ai, Junjiao Li, Yingying Yang, Mingxin Li, Cong Xue, Jing Qian, Xueli Wang, Jing Chen, Yong Li, Yanping Xiong, Jun Zhang, Tobias Stoeger, Yuhai Bi, Jianjun Chen, Zhuang Ding

**Affiliations:** ^1^Department of Veterinary Preventive Medicine, College of Veterinary Medicine, Jilin UniversityChangchun, China; ^2^Department of Food Quality and Safety, College of Food Science and Engineering, Jilin UniversityChangchun, China; ^3^CAS Key Laboratory of Special Pathogens and Biosafety, Wuhan Institute of Virology, Chinese Academy of SciencesHubei, China; ^4^Department of Veterinary Basic Medicine, College of Animal Science and Technology, Inner Mongolia University for NationalitiesTongliao, China; ^5^Hubei Wildlife Rescue, Research and Development CenterWuhan, China; ^6^Comprehensive Pneumology Center, Institute of Lung Biology and Disease (iLBD), Helmholtz Zentrum MuenchenMunich, Germany; ^7^CAS Key Laboratory of Pathogenic Microbiology and Immunology, Institute of Microbiology, Chinese Academy of SciencesBeijing, China

**Keywords:** APMV-4, intercontinental, wild birds, domestic poultry, dispersal, interspecies transmission

## Abstract

Avian paramyxovirus serotype 4 (APMV-4) is found sporadically in wild birds worldwide, and it is an economically important poultry pathogen. Despite the existence of several published strains, very little is known about the distribution, host species, and transmission of APMV-4 strains. To better understand the relationships among these factors, we conducted an APMV-4 surveillance of wild birds and domestic poultry in six provinces of China suspected of being intercontinental flyways and sites of interspecies transmission. APMV-4 surveillance was conducted in 9,160 wild birds representing seven species, and 1,461 domestic poultry in live bird markets (LMBs) from December 2013 to June 2016. The rate of APMV-4 isolation was 0.10% (11/10,621), and viruses were isolated from swan geese, bean geese, cormorants, mallards, and chickens. Sequencing and phylogenetic analyses of the 11 isolated viruses indicated that all the isolates belonging to genotype I were epidemiologically connected with wild bird-origin viruses from the Ukraine and Italy. Moreover, chicken-origin APMV-4 strains isolated from the LBMs were highly similar to wild bird-origin viruses from nearby lakes with free-living wild birds. In additional, a hemagglutination-negative APMV-4 virus was identified. These findings, together with recent APMV-4 studies, suggest potential virus interspecies transmission between wild birds and domestic poultry, and reveal possible epidemiological intercontinental connections between APMV-4 transmission by wild birds.

## Introduction

Viruses of the family *Paramyxoviridae* have been isolated from avian, terrestrial, and aquatic animals worldwide. The family *Paramyxoviridae* is divided taxonomically into two subfamilies: the *Paramyxovirinae* and *Pneumovirinae*. Members of the *Paramyxovirinae* subfamily are further classified into five genera: *Respirovirus, Morbillivirus, Rubulavirus, Henipavirus*, and *Avulavirus* (Swayne, 2013). Paramyxoviruses that have been isolated from avian species belong to the *Avulavirus* genus, and they are called avian paramyxoviruses (APMVs). APMVs have been divided into nine APMV serotypes (APMV-1 to APMV-9) based on antigenic differences revealed by hemagglutination inhibition (HI) and neuraminidase inhibition assays, as well as a full F gene sequence analysis (Swayne, 2013), and, recently, five more APMV serotypes (APMV-10 to APMV-14) have been proposed (Miller et al., 2010; Briand et al., 2012; Terregino et al., 2013; Yamamoto et al., 2015; Karamendin et al., 2016b; Thampaisarn et al., 2017).

**Figure 1:**
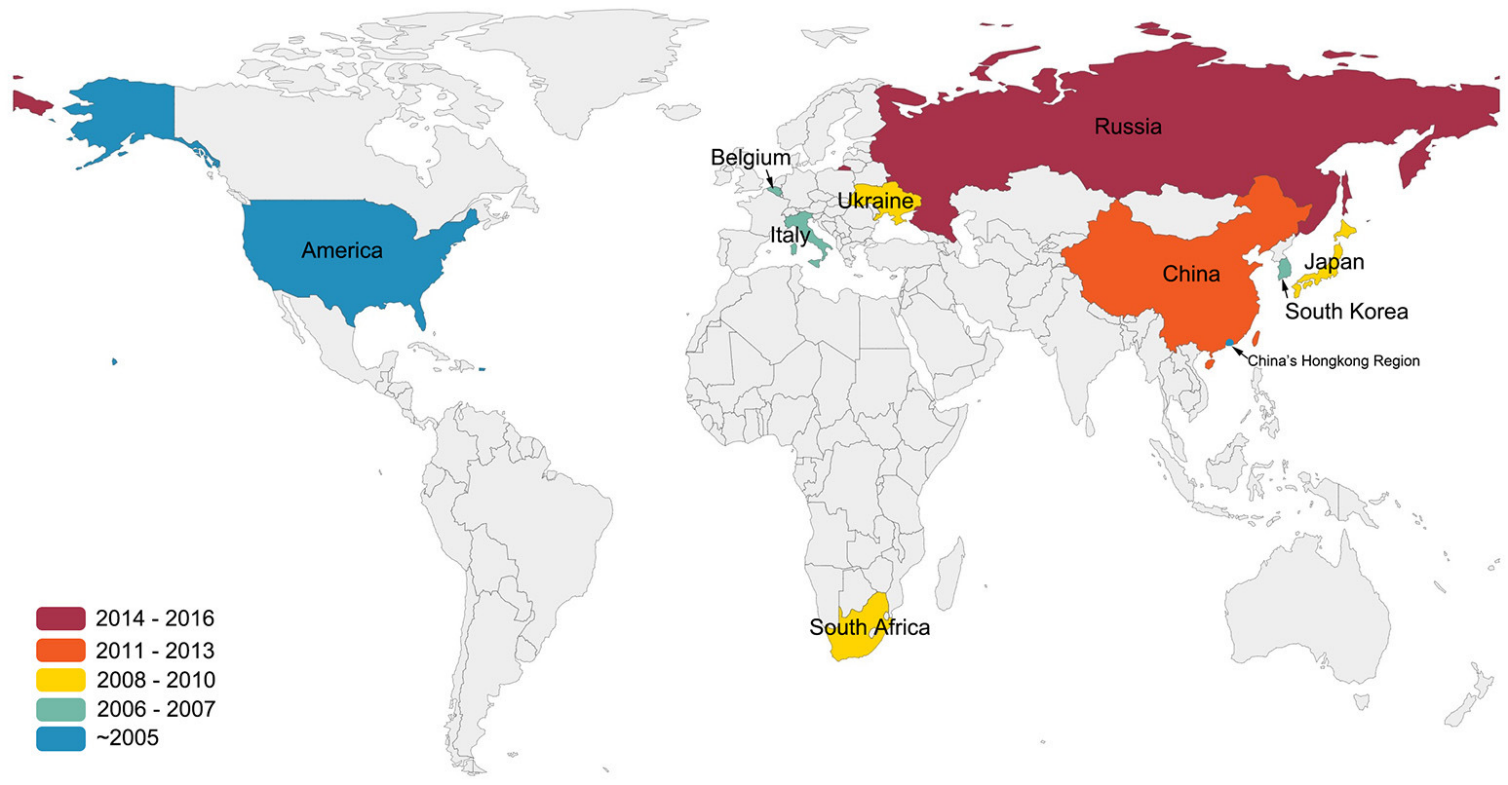
**Isolation sites and years of APMV-4 worldwide**. This figure indicates data cutoff on 25 December 2016. Colors indicate the years of the first isolation of APMV-4 in the world. All data were obtained from GenBank (http://www.ncbi.nlm.nih.gov/GenBank/).

Although APMV-1, which is synonymous with Newcastle Disease Virus (NDV), is highly pathogenic to poultry, APMV-4 is also known to cause an increase in white-shelled eggs, mild interstitial pneumonia, and catarrhal tracheitis in chickens (Warke et al., 2008; Swayne, 2013). In 1975, APMV-4/duck/Hong Kong/D3/75 became the first APMV-4 strain to be isolated in Hong Kong from a duck (Shortridge and Alexander, 1978). Wild birds, particularly waterfowl, are known reservoirs and important carriers of APMV-4 (Stanislawek et al., 2002; Karamendin et al., 2016b; Reeves et al., 2016). Meanwhile, many APMV-4 strains also have been isolated from domestic poultry, such as ducks and geese (Shortridge and Alexander, 1978; Wang et al., 2013). Very little is known about the dispersal and transmission of APMV-4 strains among wild and domestic birds, although they have been isolated worldwide (Shortridge and Alexander, 1978; Jeon et al., 2008; Nayak et al., 2008; Abolnik et al., 2012; Choi et al., 2013; Nayak et al., 2013; Wang et al., 2013; Muzyka et al., 2014; Karamendin et al., 2016a; Reeves et al., 2016; Figure 1).

The potential for AMPV-4 dispersal and transmission is high because many migratory waterfowl, such as wild ducks, wild geese, and swans, overwinter in China after migrating from Alaska, eastern Siberia, eastern Mongolia, and the Russian Far East (Bi et al., 2016). Previous studies demonstrated that wild birds are potentially capable of transmitting and spreading precursors of APMV strains to domestic poultry (Warke et al., 2008; Choi et al., 2013). Consequently, continuous and systemically surveillance of APMV-4 strains among wild birds and domestic poultry is important for providing information on viruses, as well as emerging viruses, in the field. Here, we conducted a systematical survey of APMV-4 in populations of migratory wild birds and domestic poultry from December 2013 to June 2016 in six provinces of China.

## Materials and methods

### Ethical states

All experimental protocols used in this work were reviewed and approved by the Experimental Animal Council of Jilin University, China.

### Sample collection

During December 2013 to June 2016, 1,461 tracheal or cloacal swab samples were collected from clinically healthy birds of LBMs and 9,160 fecal samples were collected from wetlands in China for APMV-4 epidemiological surveillance. The samples were collected in one province in East China (Anhui), two in Central China (Hubei and Hunan), one in Northwest China (Qinghai), one in Northeast China (Jilin), and one in North China (Neimenggu).

Samples from birds were taken using sterile swabs placed in viral transport medium containing 2,000 U/ml penicillin, 2 mg/ml streptomycin, 50 μg/ml gentamycin, 50 U/ml nystatin, and 0.5% bovine serum albumin. Samples were kept in liquid nitrogen during the fieldwork, and were stored at −80°C after return to the laboratory.

### Virus isolation

All samples were inoculated into allantoic cavities of 9 to 10-day old specific-pathogen-free (SPF) chicken embryos (Beijing Merial Vital Laboratory Animal Technology Co., Ltd., Beijing, China) and incubated 96 h at 37°C (Kim et al., 2007). Allantoic fluids from inoculated eggs were harvested either when the embryos were killed or after the two passages. The presence of the APMV-4 in allantoic fluid was confirmed by reverse transcription PCR (RT-PCR) for paramyxovirinae viruses (Tong et al., 2008, Table 2).

### RNA extraction and RT-PCR

Total RNA, including viral RNA, was extracted from allantoic fluid using Tripure (Roche, Germany) following manufacturer's recommendations. Following extraction, samples were assayed by RT-PCR for paramyxovirinae viruses (Tong et al., 2008, Table 2) using One-Step RT-PCR kit (Takara, Japan) in accordance with the manufacturer's instructions. The cycling conditions consisted of 45 min at 48°C and then an initial denaturation at 94°C for 2 min followed by 40 cycles of 94°C for 15 s, primer annealing at 48 to 50°C for 30 s, and 72°C for 30 s. A final extension was carried out at 72°C for 7 min. The short genome fragment was sequenced by an ABI 3730xl DNA analyzer (Applied Biosystems).

A BLAST search confirmed the viruses belongs to APMV-4. Then the positive for APMV-4 samples were amplified for complete F gene and HN gene. Conditions for PCR were as follows: 95°C for 3 min, followed by 35 cycles at 95°C for 1 min, 51°C for 45 s, and 72°C for 2 min 30 s, with a final extension step at 72°C for 10 min. The primer sequences used in this work are listed in Table 2. Sequencing of PCR amplicons was conducted by Major-bio Company (Shanghai, China).

### Phylogenetic analysis

Obtained nucleotide sequences were aligned using Mega 6.06 software with the sequences of 69 APMV-4 reference strains obtained from Genbank (http://www.ncbi.nlm.nih.gov/GenBank). Alignment and comparison of the nucleotide and amino acid sequences were performed using the MegAlign program in the Lasergene package (DNASTAR, Inc., Madison, WI). A maximum-likelihood tree was generated using MEGA 6.06.

## Result

**Figure 2:**
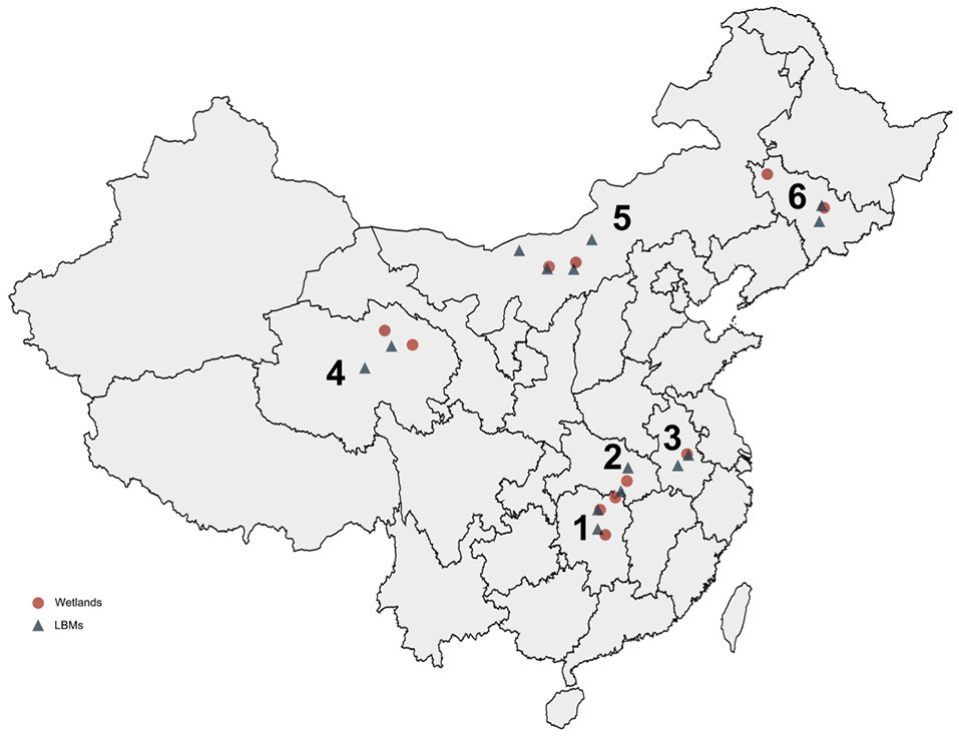
**Distribution of collected samples from wild birds and domestic poultry in China from 2013 to 2016**. The six provinces are marked by numbers: 1, Hunan; 2, Hubei; 3, Anhui; 4, Qinghai; 5, Neimenggu; 6, Jilin.

From 2013 to 2016, we conducted a virological examination of fecal samples collected from 9,160 wild birds belonging to seven species. The largest number of samples was collected from swan geese (2,384 samples), followed by the great black-headed gull (2,102 samples), and bar-headed geese (2,051 samples). In addition, 1,461 tracheal and cloacal swab samples were collected from clinically healthy chickens (878 samples) and ducks (583 samples) in 14 live bird markets (LBMs). Taxa from a range of niches were sampled, including aquatic (86%) and terrestrial (14%) avifauna. These monitoring sites covered most of the migrating birds' nesting and resting sites in China: Qinghai Lake in western China, Songhua Lake in northeastern China, Wuliangsuhai Lake in northern China, and Tungting Lake, Honghu Lake, and Chenhu Lake in central China. Detailed results of the collection sites, the numbers of biological samples collected, and the virological findings are shown in Table 1, Figure 2.

**Table 1:** **Number of samples of biological material taken from birds of different ecological groups in China from 2013 to 2016 and the result of APMV-4 isolation^a^**.

**Bird**	**No. of samples during:**			**No. (%) of samples obtained in LBMs**	**Positive samples/total No. of samples**
	**Autumn migration**	**Wintering**	**Spring migration, nesting, and post-nesting movements**		
Mallard (*Anas platyrhynchos*)	26	123	1/255		1/404 (0.25)
Swan goose (*Anser cygnoides*)	90	3/1,722	1/572		4/2,384 (0.17)
Bean goose (*Anser fabalis*)	1/34	1/916	146		2/1,096 (0.18)
Bar-headed goose (*Anser indicus*)	21	478	1552		0/2,051 (0)
Cormorant (*Phalacrocorax* sp.)	140	2/434	477		2/1,051 (0.19)
Great blackheaded Gull (*Larus ichthyaetus*)	122	426	1,554		0/2,102 (0)
Whiskered tern (*Chlidonias hybrida*)	0	24	48		0/72 (0)
Chicken (*Gallus domesticus*)				2/878	2/878 (0.23)
Duck (*Anas platyrhynchos domestica*)				0/583	0/583 (0)
Total	1/433 (0.23)	6/4,123 (0.15)	2/4,604 (0.04)	2/1,461 (0.14)	11/10,621 (0.10)

a *The results are presented as the number of samples alone or the total number/number of isolated viruses. The percentages given in parentheses represent the percentages of positive samples*.

**Table 2:** **Primers used in this study**.

**Fragment**	**Primer sequence (5′–3′)**	**Position**	**Source or references**
PNE-F1	GTGTAGGTAGIATGTTYGCNATGCARCC		Tong et al., 2008
PNE-F2	ACTGATCTIAGYAARTTYAAYCARGC		Tong et al., 2008
PNE-R	GTCCCACAAITTTTGRCACCANCCYTC		Tong et al., 2008
APMV-4_F_F4114	GGGGGTGAGCAGGAGTATGT	4,114	Choi et al., 2013
APMV-4_F_R4763	GCACCTGTGGCTATTATTGC	4,763	Choi et al., 2013
APMV-4_F_F4531	CTTTGTAACTCAAGTCCGACA	4,531	This study
APMV-4_F_R5984	GAGAACAACAATCAGACCGAT	5,984	This study
APMV-4_F_F5713	TCGCTTCAACATACAGACTGA	5,713	This study
APMV-4_F_R7385	ATCCTCCTACCGAATCGAGT	7,385	This study
APMV-4_F7046	CTCCTTTGCAGCTTAGTGTCG	7,046	This study
APMV-4_R8945	CTGAACTTCGGTGATTGCTT	8,945	This study

Only a limited number of APMV-4 epidemiological surveillance studies have been conducted previously in wild birds and domestic poultry. This may be because APMV-4 virus detection was neglected since these viruses are negligible economically impact to poultry industry. In this study, our data showed that the rate of AMPV-4 isolation in the collected samples was 0.10% (11/10,621) (Table 1), and the APMV-4 isolation rates for wild birds and domestic poultry were 0.10% (9/9,160) and 0.14% (2/1,461), respectively. Interestingly, no APMV-4 strain was isolated from ducks. Future APMV-4 monitoring studies should include more samples from ducks in LBMs as well as their susceptibility to APMV-4 viruses may differ from that of other avian species. Taken together, our data demonstrate that APMV-4 has a lower isolate rate virus (0.1%) in wild and domestic birds compared with APMV-1.

**Figure 3:**
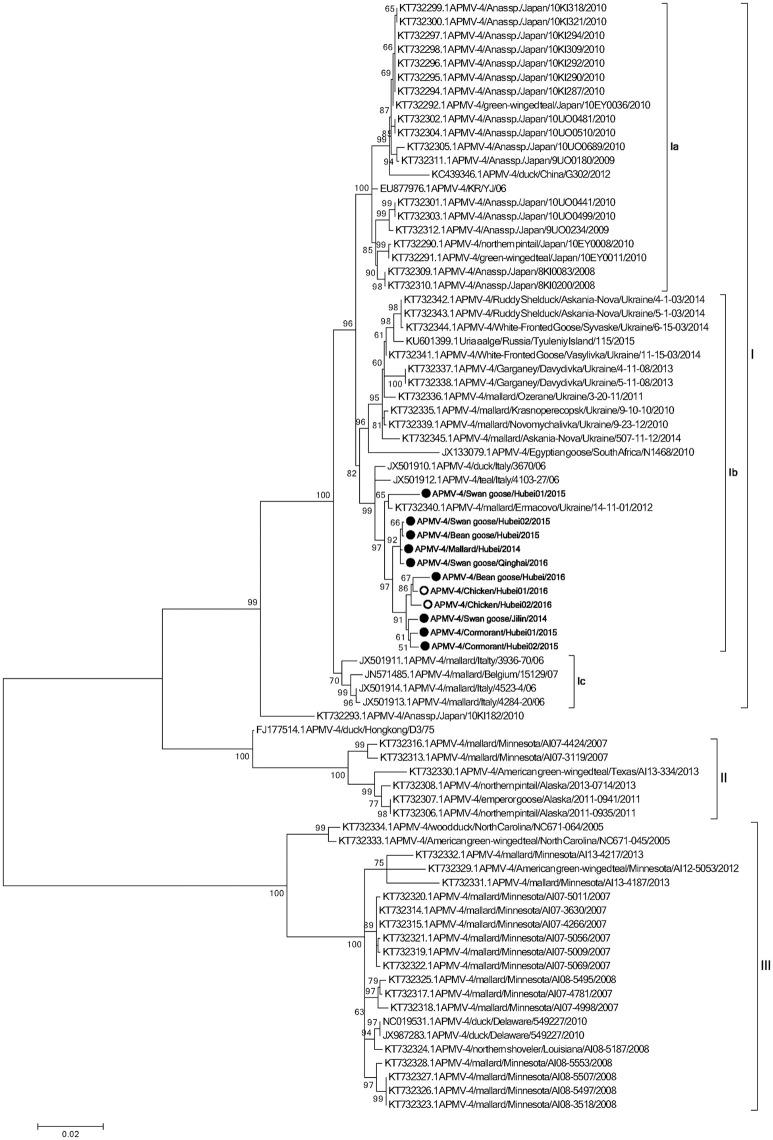
**Phylogenetic analysis of complete F gene sequences**. Wild bird- and domestic poultry-origin sequences are indicated as gray and white circles, respectively. Only bootstrap values of ≥50% are shown. The evolutionary history was inferred by using the Maximum Likelihood method based on the General Time Reversible model. The tree with the highest log likelihood (−7,547.0759) is shown. The percentage of trees in which the associated taxa clustered together is shown next to the branches. Initial tree(s) for the heuristic search were obtained by applying the Neighbor-Joining method to a matrix of pairwise distances estimated using the Maximum Composite Likelihood (MCL) approach. A discrete Gamma distribution was used to model evolutionary rate differences among sites (five categories; +G, parameter = 0.4307). The rate variation model allowed for some sites to be evolutionarily invariable ([+I], 0.0000% sites). The tree is drawn to scale, with branch lengths measured in the number of substitutions per site. The analysis involved 80 nucleotide sequences. All positions containing gaps and missing data were eliminated. There were a total of 1,694 positions in the final dataset. Evolutionary analyses were conducted in MEGA6.

To determine the genetic characteristics of the 11 isolated APMV-4 strains, we conducted sequencing and phylogenetic analyses of these isolates, and then a phylogenetic tree was constructed based on their complete F gene sequences, together with 69 representative APMV-4 F gene sequences (Figure 3). All 11 APMV-4 isolates in this study showed the typical APMV-4 sequence motif _116_DIQPR^*^F_121_ (the asterisk indicates the cleavage site of the F0 precursor protein into its F1 and F2 subunits; Table 3). Recently, a unified nomenclature and classification system of the APMV-1 (NDV) genotyping method was proposed by Diel et al. based on the mean inter-populational evolutionary distances of the full F protein, with cutoff values (>10% of the mean inter-populational evolutionary distance) to assign new genotypes (Diel et al., 2012). Therefore, 80 APMV-4 isolates, including the 11 isolates in this study, were divided into three genotypes (I, II, and III, with the distances between groups varying from 0.108 [10.8%] to 0.162 [16.2%]), and only two strains from Hong Kong and Japan (APMV-4/duck/Hong Kong/D3/75 and APMV-4/Anas sp./Japan/10KI182/2010, respectively) did not fall into these major genotypes (Figure 3; Table 4A). Moreover, the 51 genotype I strains formed three sub-genotypes, Ia, Ib, and Ic (with the distances between groups varying from 0.027 [2.7%] to 0.029 [2.9%]) (Table 4B), which were regarded previously as clades A, B, and C, respectively (Reeves et al., 2016). Together, these results clearly indicate the existence of multiple genotypes/sub-genotypes within APMV-4.

**Table 3:** **Detailed information of APMV-4s isolated from China over the period from 2013 to 2016**.

**APMV-4 strain**	**Site**	**Host**	**Year**	**Sub-genotype**	**F cleavage site**	**Accession no**.
APMV-4/Swan goose/Jilin/2014	Jilin	Swan goose	2014	Ib	DIQPRF	KY865332
APMV-4/Mallard/Hubei/2014	Hubei	Mallard	2014	Ib	DIQPRF	KY865333
APMV-4/Cormorant/Hubei01/2015	Hubei	Cormorant	2015	Ib	DIQPRF	KY865341
APMV-4/Cormorant/Hubei02/2015	Hubei	Cormorant	2015	Ib	DIQPRF	KY865342
APMV-4/Swan goose/Hubei01/2015	Hubei	Swan goose	2015	Ib	DIQPRF	KY865334
APMV-4/Bean goose/Hubei/2015	Qinghai	Bean goose	2015	Ib	DIQPRF	KY865335
APMV-4/Swan goose/Hubei02/2015	Hubei	Swan goose	2015	Ib	DIQPRF	KY865336
APMV-4/Bean goose/Hubei/2016	Hubei	Bean goose	2016	Ib	DIQPRF	KY865339
APMV-4/Chicken/Hubei01/2016	Hubei	Chicken	2016	Ib	DIQPRF	KY865338
APMV-4/Chicken/Hubei02/2016	Hubei	Chicken	2016	Ib	DIQPRF	KY865340
APMV-4/Swan goose/Qinghai/2016^a^	Qinghai	Swan goose	2016	Ib	DIQPRF	KY865337

a *This strain shows negative hemagglutination*.

**Table 4:** **Estimates of evolutionary distances between APMV-4 genotypes (A)/subgenotypes (B)^a^**.

**A**			
**Genotype**	**No. of base substitutions per site or standard error estimate**^b^		
	**I**	**II**	**III**
I		0.013	0.018
II	0.130		0.019
III	0.191	0.194	
**B**			
**Subgenotype**	**No. of base substitutions per site or standard error estimate**^c^		
	**Ia**	**Ib**	**Ic**
Ia		0.003	0.004
Ib	0.027		0.004
Ic	0.026	0.029	

a *Inferred from the complete nucleotide F gene sequence*.

b *The number of base substitutions per site from averaging over all sequence pairs between groups are shown. Standard errors are shown above the diagonal, obtained by a bootstrap procedure (500 replicates). Analyses were conducted using the Maximum Composite Likelihood model. The analysis involved 78 nucleotide sequences (I, n = 51; II, n = 6; III, n = 21). Codon positions included were I + II + III + Non-coding. All positions containing gaps and missing data were eliminated. There were a total of 1,694 positions in the final dataset. Evolutionary analyses were conducted in MEGA6*.

c *The number of base substitutions per site from averaging over all sequence pairs between groups are shown. Standard errors are shown above the diagonal, obtained by a bootstrap procedure (500 replicates). Analyses were conducted using the Maximum Composite Likelihood model. The analysis involved 51 nucleotide sequences (Ia, n = 21; Ib, n = 26; Ic, n = 4). Codon positions included were I + II + III + Non-coding. All positions containing gaps and missing data were eliminated. There were a total of 1,698 positions in the final dataset. Evolutionary analyses were conducted in MEGA6*.

To explore potential epidemiological and intercontinental connections between APMV-4 viruses and wild birds, we compared the percentages of gene sequence identities between the full F gene of the APMV-4 isolates in this study with those of reference strains. In previous studies, many APMV-4 strains were isolated from the same species in several Asian countries, including Japan (Reeves et al., 2016), South Korea (Jeon et al., 2008; Choi et al., 2013), and China (Wang et al., 2013). Interestingly, the F gene sequences of these Asian APMV-4 isolates were highly similar and clustered into sub-genotype Ia, whereas all 11 APMV-4 strains in this study clustered into sub-genotype Ib, which is closer phylogenetically to waterfowl isolates from Italy (APMV-4/teal/Italy/4103-27/06 and APMV-4/duck/Italy/3670/06) and the Ukraine (APMV-4/mallard/Ermacovo/Ukraine/14-11-01/2012) (Nayak et al., 2008; Reeves et al., 2016), suggesting a close phylogenetic relationship between the European and recent Asian isolates. However, no sub-genotype Ia APMV-4 isolate was obtained in this study. Highly similar sub-genotype Ib APMV-4 isolates from distinct species in different cities of Europe and Asian clearly indicate that the virus can be transmitted intercontinentally by wild birds.

To determine the potential epidemiological connections between wild birds and domestic poultry, we compared the percent similarities of the full F gene nucleotide sequences of eight APMV-4 strains isolated from Hubei Province. Two of the eight APMV-4 isolates (APMV-4/Chicken/Hubei01/2016 and APMV-4/Chicken/Hubei02/2016) were isolated from chickens in LBMs near lakes in 2016, and their complete F gene sequence was highly similar to that of the wild bird-origin APMV-4 strain APMV-4/mallard/Ermacovo/Ukraine/14-11-01/2012 (nucleotide sequence homologies of 98.7%). Moreover, the complete F genes of these two chicken-origin APMV-4 strains shared 99.1–99.3% nucleotide similarities with a wild bird-origin strain from LBMs near Chenhu Lake (APMV-4/Bean goose/Hubei/2016), suggesting that the virus can be transmitted between wild birds and domestic poultry and that previously detected APMV-4 isolates from wild birds will likely be detected from domestic poultry in the future, and vice versa.

Earlier findings showed that the APMV-4 (APMV4/duck/China/G302/2012) strain cannot hemagglutinate the red blood cells of chickens, ducks, geese, and humans (type O) (Wang et al., 2013). In this study, the hemagglutination (HA)-negative virus APMV-4/Swan goose/Qinghai/2016 strain displayed the same characteristic; however, we could not identify distinguishing characteristics of the nucleotide and amino acid sequences of the HA-neuraminidase (HN) gene between HA-negative and HA-positive strains (Table S1). The NRKSCS motif has been identified in the HN protein in other serotypes of avian paramyxoviruses, and it is thought to be involved in sialic acid binding (Mirza et al., 1994; Xiao et al., 2009). In line with other APMV-4 isolates, this motif was also present at amino acid positions 240–245 in the HN protein of all 11 APMV-4 isolates in this study. However, the APMV-4/Swan goose/Qinghai/2016 strain recovered the ability to hemagglutinate red blood cells when it was pretreated with 1% trypsin for 1 h (data not shown). Further studies will be necessary to improve our understanding of HA-negative APMV-4 strains in the field.

## Discussion

The main goal of this study was to increase our understanding of the ecology of APMV-4 strains in wild and domestic birds. To obtain a more accurate estimate of the transmission potential of each bird species, virus isolation from swabs or feces was conducted, instead of more sensitive, but less reliable, nucleic-acid-based methods. Moreover, the virus isolation allowed us to characterize the isolates.

From 2013 to 2016, viruses from nine of the 11 APMV-4 strains were isolated from four different species of wild birds: swan geese, bean geese, cormorants, and mallards. Additionally, two viruses were obtained from chickens in LBMs, and the rate of APMV-4 isolation was 0.1%. Similar data were obtained by other researchers who conducted monitoring studies of waterfowl and migratory birds and identified APMV-4 strains (Choi et al., 2013; Muzyka et al., 2014; Karamendin et al., 2016a). In Kazakhstan, the prevalence of APMV-4 infections among wild birds was 0.18% from 2002 to 2013 (Karamendin et al., 2016a). In the Azov–Black Sea region of the Ukraine, in addition to APMV-1 and -6 strains, several APMV-4 strains were isolated from migratory birds. In these cases, the prevalence of APMV-4 infection was 0.08% (Muzyka et al., 2014).

Our data confirm that the prevalence of APMV-4 infection in wild birds varies with the environment and the characteristics of the life cycles of birds, with the highest rates of infection detected in autumn. During the autumn migration, the rate of APMV-4 isolation in wild waterfowl and birds of different species was 0.23%, while the rate of APMV-4 isolation from wild birds during winter was 0.15%. In contrast, the APMV-4 isolate rate was 0.04% in wild birds during the spring migration, nesting, and after nesting movements. Environmental conditions (temperature changes, the presence of snow cover during winter, food availability, and other factors) contribute to the formation of large groups of wild birds in a small area, which significantly increases the probability of direct contact between birds of different species and from different geographic regions.

The complete F protein-encoding gene is considered as the main target for genotyping and molecular epidemiological investigations of APMV, APMV-4 recently was suggested to classify into five clades (A to E), based on the F gene sequences of viruses isolated from different continents (Reeves et al., 2016). However, a unified nomenclature and classification system of the APMV-1 genotyping method, based on mean inter-populational evolutionary distances of the full F protein, with cutoff values (>10% of the mean inter-populational evolutionary distance) to assign new genotypes (Diel et al., 2012), will provide a more scientific and rational genotyping method for molecular epidemiological investigations of APMV-2 to -14 strains. Therefore, at least three genotypes (I, II, and III) exist within APMV-4, based on the mean inter-populational evolutionary distances of the full F protein.

Based on the existing literature of APMV-4 and our current data, viruses of genotype I have been isolated mainly from Old World countries (Europe, Asia, and Africa), whereas genotypes II and III comprise viruses originating from waterfowl from the USA. The highly homologous genotype I APMV-4 strains, which were isolated from the Old World and differ significantly from those of the American strains, indicate that there may be a barrier between the trans-Atlantic and trans-Pacific spreading of APMV-4, and even other viruses, by wild birds. In addition, the APMV-4/duck/Hong Kong/D3/75 strain is closely related to genotype II, which comprises North American strains. However, we cannot exclude the possibility of viral transmission between these continents in the future.

The phylogenetic analysis data in this study indicated that all 11 APMV-4 viruses share a high level of identity with viruses from other geographical regions in Europe, such as the Ukraine and Italy. This might be explained in terms of the migration of wild birds into these regions from Europe, since these regions are in the Black Sea/Mediterranean and Central Asian flyways, which provide an opportunity for APMV-4 transmission from Europe to Asia, and vice versa, via stopover wetland sites. Thus, these ecological features provide an opportunity for the intercontinental exchange of APMV-4 strains between Asia and Europe, while the East Asian Australian flyway may not contribute to the spread of APMV-4, compared with that of APMV-1 and avian influenza viruses (AIV) (Kou et al., 2009; Kim et al., 2011, 2012; Zhang et al., 2015).

Hemagglutination and HI assays are the traditional methods to detect APMV in many laboratories. Identifying emerging HA-negative strains that are not detected by conventional diagnostic assays is critical to continuously update surveillance systems, comprising diagnostic assays, biosecurity measures, and research, to protect domestic poultry and wild birds worldwide.

In summary, our study shows that APMV-4 is found sporadically in migratory wild birds and clinically healthy domestic poultry in China. Because Asian, Black Sea/Mediterranean, Alaska, eastern Siberia, eastern Mongolia, and the Russian Far East are connected by the migratory routes of the species analyzed here, our data should generally be applicable to Asian APMV-4 strains. Efforts are needed to restrict contacts between wild birds and domestic poultry, as these hosts appear to continuously exchange APMV-4 strains. Import, trade, and bird shows are other events that easily allow the spread of new strains to susceptible populations, unless strict control measures are applied. The APMV-4 isolates in this study are related to viruses from other geographical regions, and their presence suggests the potential risk of APMV-4 being brought into the country, possibly leading to bird infections. It also underlines the importance of constant surveillance for APMV-4 strains among wild birds and domestic poultry in China to explore the possible introduction of new genetic variants from other geographic regions.

## Author contributions

RY, PZ, JiaC, and ZD designed the study. RY and PZ drafted the manuscript and analyzed the data. RY, PZ, XL, YC, ZT, LA, JL, YY, ML, CX, JQ, XW, TS, JinC, YL, YX, JZ, YB, and JiaC collected clinical samples. RY, PZ, YC, ZT, ML, JiaC, and ZD carried out experiments. All authors read and approved the final manuscript.

### Conflict of interest statement

The authors declare that the research was conducted in the absence of any commercial or financial relationships that could be construed as a potential conflict of interest.
