# Supplementary material for: Dispersal and Transmission of Avian Paramyxovirus Serotype 4 among Wild Birds and Domestic Poultry
Source: Front Cell Infect Microbiol. 2017 May 26;7:212. doi: 10.3389/fcimb.2017.00212 (PMC5445105; doi:10.3389/fcimb.2017.00212)
Supplement: Supplementary file 1 [file Table1.DOCX]

**Table S1 The amino acid sequences (A) and nucleotide sequences (B) of hemagglutination-negative strain in comparison to the corresponding regions of hemagglutination-positive strains. Full-length HN protein sequences of these viruses were aligned using the Clustal W program, version 1.83, with default settings.**

**A**

| **Strain** | **Amino acid sequences (1-100)** |
| --- | --- |
| APMV-4/Swan goose/Qinghai/2016^a^ | **ＭＥＧＳＲＤＮＬＴＶＤＤＥＬＫＴＴＷＲＬＡＹＲＶＶＳＬＬＬＭＶＳＡＬＶＩＳＩＶＩＬＴＲＤＮＳＱＳＩＩＴＡＩＫＱＳＳＤＡＤＳＫＷＱＴＧＩＥＧＫＩＴＳＩＭＴＤＴＬＤＴＲＮＡＡＬＬＨＩＰＬＱＬＮＴＬＥＡＮＬ** |
| APMV-4/Swan goose/Jilin/2014 | **―――――――――――――――――――――――――――――――――――――――――――――――――――――Ｎ――――――――――――――――――――――――――――――――――――――――――――――** |
| APMV-4/Bean goose/Hubei/2015 | **―――――――――――――――――――――――――――――――――――――――――――――――――――――Ｎ――――――――――――――――――――――――――――――――――――――――――――――** |
| APMV-4/Swan goose/Hubei02/2015 | **―――――――――――――――――――――――――――――――――――――――――――――――――――――Ｎ――――――――――――――――――――――――――――――――――――――――――――――** |

| **Strain** | **Amino acid sequences (101-200)** |
| --- | --- |
| APMV-4/Swan goose/Qinghai/2016^a^ | **ＬＳＡＬＧＧＮＴＧＩＧＰＧＤＬＥＨＣＲＮＰＶＨＤＴＡＹＬＨＧＶＮＲＬＬＩＮＱＴＡＤＹＴＡＥＧＰＬＤＨＶＮＦＩＰＡＰＶＴＴＴＧＣＴＲＩＰＳＦＳＶＳＳＳＩＷＣＹＴＨＮＶＩＥＴＧＣＮＤＨＳＧＳＮＱＹＩＳＭＧ** |
| APMV-4/Swan goose/Jilin/2014 | **――――――――――――――――――――――――――――――――――――――――――――――――――――――――――――――――――――――――――――――――――――――――――――――――――――** |
| APMV-4/Bean goose/Hubei/2015 | **―――――――――――――――――――Ｙ――――――――――――――――――――――――――――――――――――――――――――――――――――――――――――――――――――――――――――――――** |
| APMV-4/Swan goose/Hubei02/2015 | **―――――――――――――――――――Ｙ――――――――――――――――――――――――――――――――――――――――――――――――――――――――――――――――――――――――――――――――** |

| **Strain** | **Amino acid sequences (201-300)** |
| --- | --- |
| APMV-4/Swan goose/Qinghai/2016^a^ | **ＶＩＫＲＡＧＮＧＬＰＹＦＳＴＶＶＳＫＹＬＴＤＧＬＮＲＫＳＣＳＶＡＡＧＳＧＨＣＹＬＬＣＳＬＶＳＥＰＥＰＤＤＹＶＳＰＤＰＴＰＭＲＬＧＶＬＴＷＤＧＳＹＴＥＱＶＶＰＥＲＩＦＫＮＩＷＳＡＮＹＰＧＶＧＳＧＡＩＶＧ** |
| APMV-4/Swan goose/Jilin/2014 | **――――――――――――――――――――――――――――――――――――――――――――――――――――――――――――――――――――――――――――――――――――――――――――――――――――** |
| APMV-4/Bean goose/Hubei/2015 | **――――――――――――――――――――――――――――――――――――――――――――――――――――――――――――――――――――――――――――――――――――――――――――――――――――** |
| APMV-4/Swan goose/Hubei02/2015 | **――――――――――――――――――――――――――――――――――――――――――――――――――――――――――――――――――――――――――――――――――――――――――――――――――――** |

| **Strain** | **Amino acid sequences (301-400)** |
| --- | --- |
| APMV-4/Swan goose/Qinghai/2016^a^ | **ＮＫＶＬＦＰＦＹＧＧＶＲＮＧＳＴＰＥＶＶＮＲＧＲＹＹＹＩＱＤＰＮＤＹＣＰＤＰＬＱＤＱＩＬＲＡＥＱＳＹＹＰＴＲＦＧＲＲＭＶＭＱＧＶＬＡＣＰＶＳＮＮＳＴＶＡＳＱＣＱＳＹＹＦＮＮＳＬＧＦＩＧＡＥＳＲＩＹＹＬ** |
| APMV-4/Swan goose/Jilin/2014 | **――――――――――――――――――――――――――――――――――――――――――――――――――――――――――――――――――――――――――Ｉ―――――――――――――――――――――――――** |
| APMV-4/Bean goose/Hubei/2015 | **―――――――――――――――――――Ｍ――――――――――――――――――――――――――――――――――――――――――――――――――――――――――――――――――――――――――――――――** |
| APMV-4/Swan goose/Hubei02/2015 | **―――――――――――――――――――Ｍ――――――――――――――――――――――――――――――――――――――――――――――――――――――――――――――――――――――――――――――――** |

| **Strain** | **Amino acid sequences (401-500)** |
| --- | --- |
| APMV-4/Swan goose/Qinghai/2016^a^ | **ＮＧＮＩＹＬＹＱＲＳＳＳＷＷＰＨＰＱＩＹＬＬＤＳＲＩＡＳＰＧＴＱＮＩＤＳＧＶＮＬＫＭＬＮＶＴＶＩＴＲＰＳＳＧＦＣＮＳＱＳＲＣＰＮＤＣＬＦＧＶＹＳＤＩＷＰＬＳＬＴＳＤＳＩＦＡＦＴＭＹＬＱＧＫＴＴＲＩＤＰ** |
| APMV-4/Swan goose/Jilin/2014 | **――――――――――――――――――――――――――――――――――――――――――――――――――――――――――――――――――――――――――――――――――――――――――――――――――――** |
| APMV-4/Bean goose/Hubei/2015 | **――――――――――――――――――――――――――――――――――――――――――――――――――――――――――――――――――――――――――――――――――――――――――――――――――――** |
| APMV-4/Swan goose/Hubei02/2015 | **――――――――――――――――――――――――――――――――――――――――――――――――――――――――――――――――――――――――――――――――――――――――――――――――――――** |

| **Strain** | **Amino acid sequences (501-566)** |
| --- | --- |
| APMV-4/Swan goose/Qinghai/2016^a^ | **ＡＷＡＬＦＳＮＨＡＩＧＨＥＡＲＬＦＮＫＥＶＳＡＡＹＳＴＴＴＣＦＳＤＴＩＱＮＱＶＹＣＬＳＩＬＥＶＲＳＥＬＬＧＡＦＫＩＶＰＦＬＹＲＶＬ＊** |
| APMV-4/Swan goose/Jilin/2014 | **――――――――――――――――――――――――――――――――――――――――――――――――――――――――――――――――――** |
| APMV-4/Bean goose/Hubei/2015 | **――――――――――――――――――――――――――――――――――――――――――――――――――――――――――――――――――** |
| APMV-4/Swan goose/Hubei02/2015 | **――――――――――――――――――――――――――――――――――――――――――――――――――――――――――――――――――** |

**B**

| **Strain** | **Nucleotide sequences** |
| --- | --- |
| APMV-4/Swan goose/Qinghai/2016^a^ | 1-161 A 163-357 AAC 361-957 G 959-1122 GTG 1126-1698 |
| APMV-4/Swan goose/Jilin/2014 | 1-161 C 163-357 AAC 361-957 G 959-1122 ATT 1126-1698 |
| APMV-4/Bean goose/Hubei/2015 | 1-161 C 163-357 TAT 361-957 A 959-1122 GTG 1126-1698 |
| APMV-4/Swan goose/Hubei02/2015 | 1-161 C 163-357 TAT 361-957 A 959-1122 GTG 1126-1698 |

**^a^** This strain shows negative hemagglutination
